# Necrotizing scleritis resistant to biologics successfully managed with the JAK inhibitor upadacitinib: a case report

**DOI:** 10.1186/s12348-026-00580-3

**Published:** 2026-03-30

**Authors:** Tomoyuki Oyama, Shunsaku Nakai, Kenji Miyao, Masaya Imazeki, Masaru Takeuchi

**Affiliations:** Department of Ophthalmology, National Defense Medical College Hospital, 3-2 Namiki Tokorozawa Saitama, Tokorozawa, 359-8513 Japan

**Keywords:** Necrotizing scleritis, Noninfectious uveitis, Biologic resistance, JAK inhibitor, Rheumatoid arthritis

## Abstract

**Background:**

Necrotizing scleritis is a rare but severe ocular inflammatory disorder, frequently associated with systemic autoimmune diseases such as rheumatoid arthritis (RA). Although biologic agents targeting cytokines such as TNF-α and IL-6 have improved disease outcomes, some patients remain refractory to these treatments. We describe a case of necrotizing scleritis resistant to multiple biologic agents that responded favorably to the Janus kinase (JAK) inhibitor upadacitinib.

**Case presentation:**

A 43-year-old woman with rheumatoid arthritis presented with bilateral conjunctival injection and left ocular pain. Despite systemic corticosteroid therapy (30 mg/day) and switching between biologics, ocular inflammation and scleral thinning continued to progress. Consequently, oral administration of the selective JAK1 inhibitor upadacitinib (15 mg/day) was initiated. Two months after treatment, ocular pain and redness resolved completely, with marked regression of nodular inflammation and stabilization of scleral thinning. Systemic corticosteroids were successfully tapered to 5 mg/day, and the patient remained relapse-free for six months without any adverse events.

**Conclusion:**

Upadacitinib, through selective inhibition of the JAK-STAT signaling pathway, provided rapid and sustained control of inflammation in a case of necrotizing scleritis unresponsive to biologic agents. Although this observation suggests that JAK inhibitors may represent a potential therapeutic option for refractory ocular inflammation, further studies are required to clarify their long-term efficacy and safety in patients with scleritis.

## Introduction

Scleritis is a severe, vision-threatening inflammation of the scleral tissue that may lead to significant structural damage if inadequately treated [1]. Watson and Hayreh first categorized scleritis into diffuse, nodular, necrotizing, and posterior forms, with necrotizing scleritis representing the most aggressive subtype [2]. In a Japanese population, 69% had diffuse anterior scleritis, 11% had nodular anterior scleritis, 10% had necrotizing anterior scleritis and 11% had posterior scleritis [3]. It is often associated with systemic autoimmune disorders such as rheumatoid arthritis (RA) and anti-neutrophil cytoplasmic antibody (ANCA)-associated vasculitis [4]. This form is characterized by localized tissue necrosis, scleral thinning, and a high risk of perforation. Despite its rarity, necrotizing scleritis demands prompt recognition and intensive therapy due to its potential for irreversible visual loss [5].

Rheumatoid arthritis–associated scleritis accounts for a considerable subset of noninfectious scleritis cases [5]. Approximately 2% of patients with rheumatoid arthritis develop scleritis, and rheumatoid arthritis accounts for 8–15% of all scleritis cases [6]. The pathogenesis involves immune complex deposition, complement activation, and infiltration of autoreactive T lymphocytes and macrophages, which release proinflammatory cytokines such as tumor necrosis factor-α (TNF-α), interleukin-1β (IL-1β), and interleukin-6 (IL-6) [7, 8]. These inflammatory mediators activate fibroblasts and matrix metalloproteinases, resulting in progressive scleral degradation.

Corticosteroids remain the first-line therapy, followed by immunosuppressive agents such as methotrexate, cyclophosphamide, or azathioprine. Over the past two decades, biologic agents targeting specific cytokines have revolutionized the management of refractory scleritis. B-cell–depleting therapy with the anti-CD20 monoclonal antibody rituximab has demonstrated efficacy in patients with refractory scleritis [9]. Anti–TNF-α agents such as infliximab, adalimumab, and certolizumab, as well as the anti–IL-6 antibody tocilizumab, have been also shown to control inflammation and prevent relapses [6, 10–12]. However, a proportion of patients remains unresponsive or develops secondary resistance, possibly due to redundancy in cytokine signaling or anti-drug antibody formation. This therapeutic challenge has spurred the exploration of new molecular targets acting downstream of cytokine receptors.

Janus kinase (JAK) inhibitors represent a novel class of small-molecule agents that interfere with intracellular cytokine signaling. By inhibiting JAK1, JAK2, JAK3, or TYK2, these drugs block the phosphorylation of STAT proteins, thereby attenuating transcription of proinflammatory genes [13, 14]. Among them, upadacitinib—a selective JAK1 inhibitor—has demonstrated efficacy in patients with RA refractory to biologic disease-modifying antirheumatic drugs (bDMARDs) [15, 16]. Beyond rheumatologic conditions, several reports have indicated the efficacy and safety of JAK inhibitors in controlling ocular inflammatory relapses, confirming their value as a treatment option for patients with non-infectious inflammatory ocular diseases that are resistant to conventional therapies [17, 18]. 

In this context, we report a case of necrotizing scleritis associated with rheumatoid arthritis that was refractory to multiple biologic therapies but achieved complete remission with upadacitinib. This case further supports the emerging evidence that JAK inhibitors represent a valuable addition to the therapeutic armamentarium for refractory ocular inflammatory diseases.

## Case presentation

A 43-year-old woman with a 25-year history of rheumatoid arthritis (RA) presented with redness, pain, and foreign-body sensation in the left eye for one month. She had previously undergone LASIK surgery and had experienced mild episodes of scleritis several years earlier that responded to corticosteroids. Her RA had been well controlled with oral prednisolone (5 mg/day) and subcutaneous certolizumab. She had no history of ocular trauma, infection, or systemic vasculitis.

**Fig. 1:**
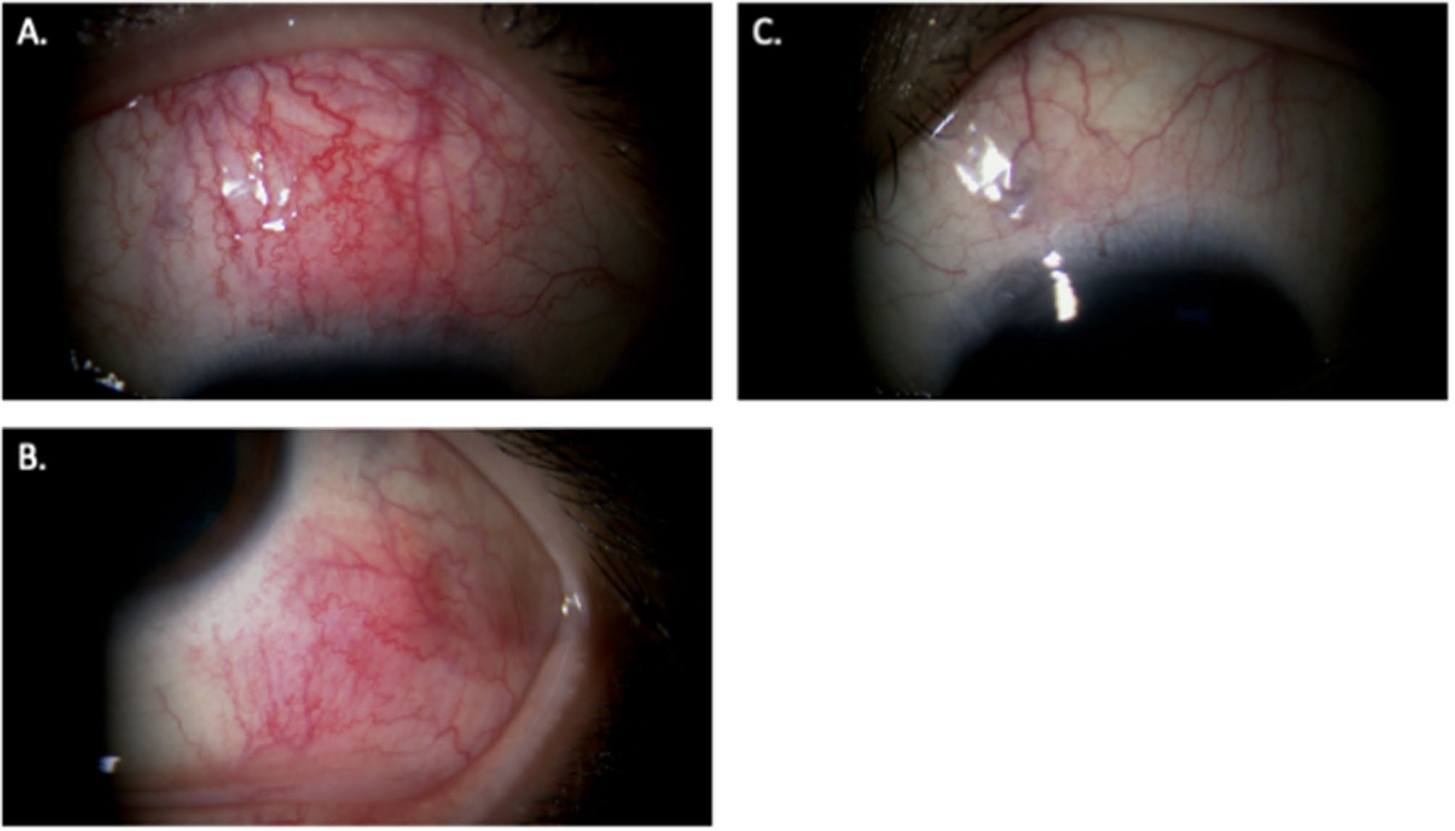
Slit-lamp photograph at initial presentation. Nodular ciliary injection was observed in the superior area of the right eye (**A**) and in the inferotemporal area of the left eye (**B**), together with scleral thinning in the superonasal area of the left eye (**C**)

At presentation, her best-corrected visual acuity was 1.5 in both eyes, and intraocular pressure was 12 mmHg in the right eye and 9 mmHg in the left eye. Slit-lamp examination revealed nodular thickening with ciliary injection in the superior area of the right eye and in the inferotemporal area of the left eye, accompanied by localized scleral thinning in the superonasal area of the left eye (Fig. 1).

The conjunctival vessels were tortuous and immobile. The cornea and anterior chamber were otherwise unremarkable, and fundus examination showed no posterior segment involvement. Laboratory investigations demonstrated elevated C-reactive protein (1.8 mg/dL) and erythrocyte sedimentation rate (41 mm/h), consistent with active systemic inflammation. Tests for infectious etiologies, including syphilis, tuberculosis, and sarcoidosis, were negative.

**Fig. 2:**
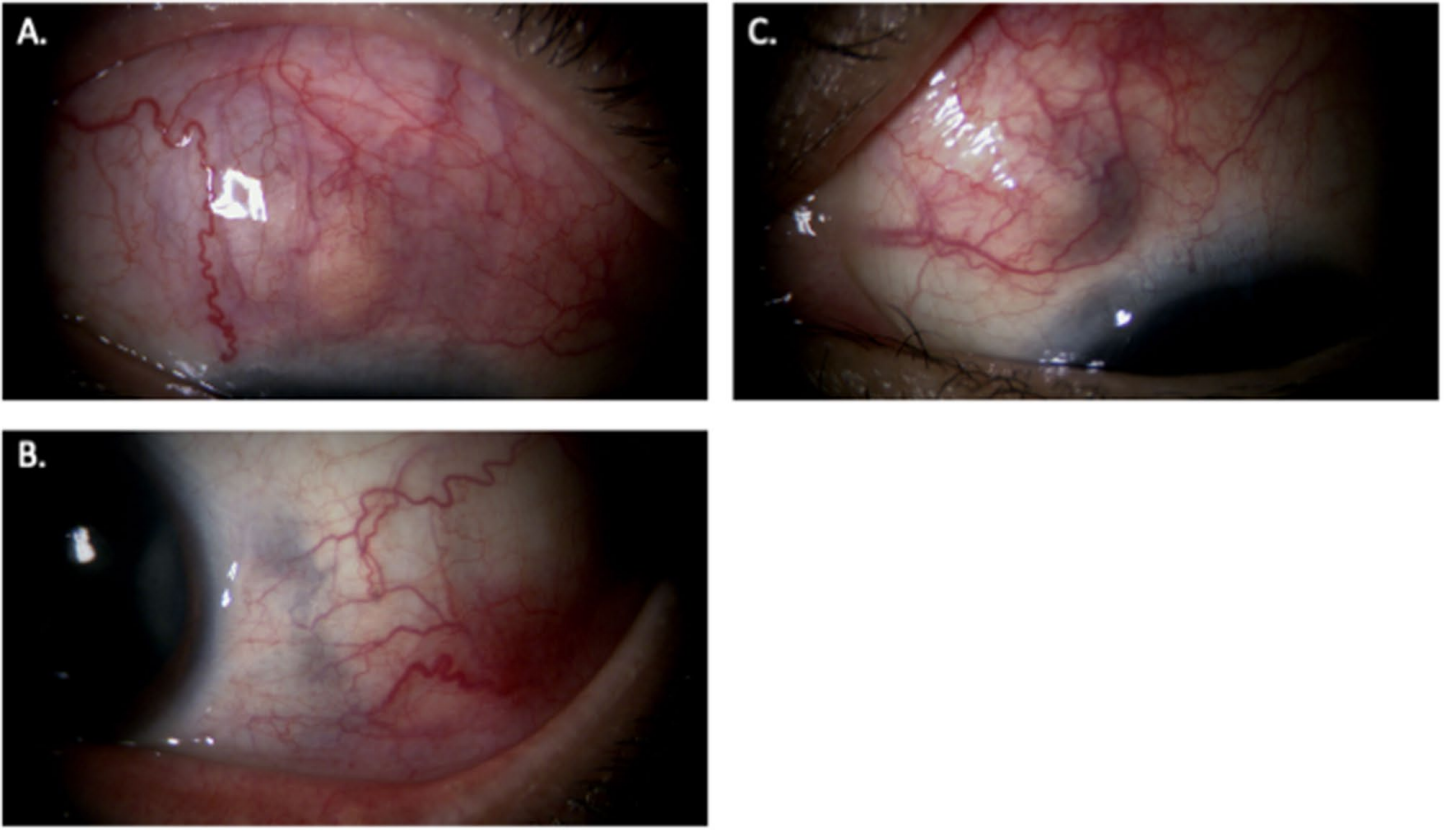
Findings just before initiation of upadacitinib under tocilizumab therapy. Despite administration of tocilizumab, nodular ciliary injection and thinning in the superior area of the right eye (**A**), as well as scleral thinning in the inferotemporal (**B**) and superonasal (**C**) regions of the left eye, further worsened

The patient’s oral prednisolone was increased to 30 mg/day, and certolizumab was continued. Despite this regimen, her ocular pain and nodular ciliary injection in the superior region of the right eye and the inferotemporal region of the left eye further worsened, and scleral thinning progressed in the left eye within four weeks. Certolizumab was replaced with etanercept based on the clinical judgment of a rheumatology specialist who had been managing the patient’s rheumatoid arthritis prior to the onset of scleritis, leading to temporary improvement in redness and pain in the right eye, while scleral thinning in the inferotemporal and superonasal regions of the left eye showed mild progression. Despite insufficient improvement, oral prednisolone was tapered at the patient’s strong request by 5 mg/day every two weeks, leading to worsening nodular ciliary injection with thinning in the right eye and progression of scleral thinning in the left eye at a dose of 20 mg/day. Etanercept was subsequently switched to the anti–IL-6 monoclonal antibody tocilizumab. Despite treatment with tocilizumab, nodular ciliary injection and thinning in the superior region of the right eye, together with scleral thinning in the inferotemporal and superonasal regions of the left eye, further progressed (Fig. 2). Given the refractory course despite multiple biologic therapies, oral upadacitinib, a selective JAK1 inhibitor, was introduced at a dose of 15 mg/day.

**Fig. 3:**
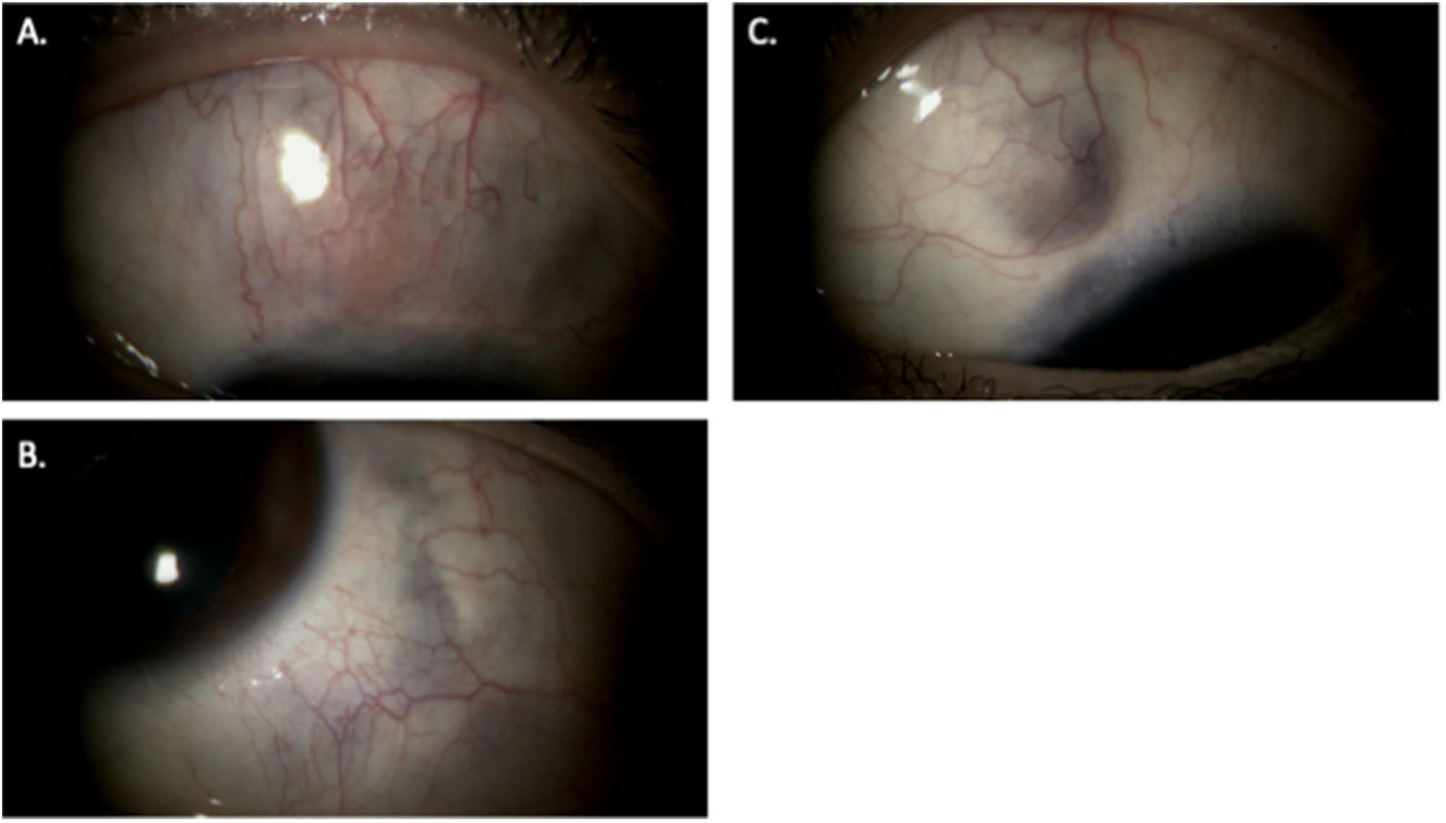
Two months after initiation of upadacitinib therapy. The nodular ciliary injection in the superior area of the right eye (**A**) resolved, and no further progression of scleral thinning was observed in the superior region of the right eye (**A**), the inferotemporal region of the left eye (**B**), or the superonasal region of the left eye (**C**)

Within two months of initiating upadacitinib, ocular pain resolved completely, and conjunctival hyperemia and nodular thickening markedly improved (Fig. 3).

The scleral thinning stabilized, and no further progression was observed. Systemic corticosteroids were gradually tapered to 5 mg/day without recurrence. During the six-month follow-up, no adverse events—including hepatic dysfunction, cytopenia, or infection—were observed. The patient’s visual acuity remained stable at 1.5 in both eyes.

## Discussion

Necrotizing scleritis represents the most severe phenotype within the scleritis spectrum, characterized by granulomatous inflammation, scleral necrosis, and a risk of globe perforation [2]. The disease often arises in the context of systemic autoimmune disorders, particularly RA, where chronic cytokine-driven inflammation contributes to both articular and ocular pathology [5, 6, 19]. While conventional immunosuppressants and biologic agents have greatly improved visual outcomes, a small but challenging subset of patients remains refractory to these therapies. This case highlights the successful use of the selective JAK1 inhibitor upadacitinib in treating necrotizing scleritis refractory to multiple biologic agents.

The clinical course of the present case suggests that blockade of a single cytokine pathway may be insufficient in certain patients with severe RA-associated necrotizing scleritis. Although anti-TNF agents are effective in many cases, some patients exhibit primary non-response or secondary loss of efficacy, a phenomenon often referred to as class failure. In addition, paradoxical inflammatory reactions, including the induction or worsening of ocular inflammation under TNF inhibition, have been reported in patients with immune-mediated diseases [19, 20]. These observations support the concept that the inflammatory milieu in refractory necrotizing scleritis may be driven by multiple cytokine pathways, including not only TNF-α but also IL-6, IFN-γ, and other JAK-STAT–dependent cytokines, rendering single-cytokine blockade insufficient.

JAK inhibitors modulate immune responses by blocking the phosphorylation of STAT proteins downstream of cytokine receptors [17, 20]. This mechanism allows for the simultaneous suppression of multiple cytokine pathways, including IL-2, IL-6, IL-12, IL-23, and interferon (IFN)-γ, which are implicated in the pathogenesis of autoimmune scleritis [13, 21, 22]. Upadacitinib, a second-generation selective JAK1 inhibitor, provides potent and targeted cytokine inhibition while minimizing off-target toxicity associated with pan-JAK blockade [13, 23–25]. In phase 3 trials for rheumatoid arthritis, upadacitinib demonstrated superior efficacy compared to abatacept in patients refractory to biologic therapies, underscoring its role in difficult-to-treat immune-mediated diseases [24].

Mechanistically, JAK inhibition may address the cytokine redundancy that underlies biologic resistance. Liu et al. demonstrated that JAK1 blockade suppresses IL-6 and IFN-γ signaling, reducing Th1 and Th17 differentiation—key drivers of scleritis pathogenesis [26]. Similarly, Yan et al. identified TNF and IL6 as hub genes in bioinformatic analyses of scleritis-related inflammation, supporting the concept that targeting downstream JAK-STAT signaling may provide broader control [27]. Furthermore, upadacitinib’s oral administration offers practical advantages in chronic management, improving adherence and patient quality of life compared to injectable biologics.

Given the complex cytokine network underlying scleritis, targeting multiple signaling pathways simultaneously may achieve more effective disease control than blockade of a single cytokine. Paley et al. reported that tofacitinib was effective in controlling refractory uveitis and scleritis unresponsive to conventional and biologic therapies, and Kim et al. further demonstrated that long-term tofacitinib administration induced sustained remission in patients with systemic autoimmune disease–associated refractory scleritis. On the other hand, Baquet-Walscheid et al. described a case of severe bilateral scleritis successfully treated with upadacitinib. Moreover, among patients with non-infectious ocular inflammation, upadacitinib demonstrated marked efficacy in several cases of refractory scleritis, leading to clinical remission where conventional immunosuppressants and biologic agents had failed [17]. These findings align with the present case, in which upadacitinib achieved dramatic improvement after the failure of multiple cytokine-targeted biologics.

Based on currently available evidence, JAK inhibitors may be particularly suitable for patients with refractory, biologic-resistant, necrotizing or vasculitic subtypes of scleritis, especially those associated with systemic autoimmune diseases such as RA. These phenotypes are thought to be driven by Th1/Th17-skewed and interferon-related immune responses, as has been described in rheumatoid arthritis and other systemic autoimmune diseases [7, 8, 22, 23], in which multi-cytokine inhibition may be more effective than single-target biologics. However, it should be emphasized that this assumption is based on limited case reports and small case series, and definitive predictors of response remain to be established.

Despite these advantages, JAK inhibitors are not without risks. Reported adverse events include an increased susceptibility to infections, elevations in hepatic enzymes, and thromboembolic complications such as deep vein thrombosis and pulmonary embolism [28, 29]. Kraev et al. emphasized the importance of careful safety monitoring, including regular assessment of liver function and cardiovascular risk, particularly in patients with underlying hepatic or cardiovascular comorbidities [28]. Pharmacovigilance analyses have also identified infections and thromboembolic events among the most frequently reported safety signals associated with JAK inhibitor therapy [29]. In our case, upadacitinib was well tolerated over six months of therapy, with no laboratory or clinical complications. The absence of relapse during corticosteroid tapering further supports its durable anti-inflammatory efficacy.

The main limitation of this report is its single-case nature. The clinical response observed in this patient may not be generalizable to all patients with necrotizing scleritis. In addition, the follow-up period was relatively short, and longer observation is required to determine the durability of treatment response and the long-term safety of upadacitinib. Another limitation is that treatment decisions regarding biologic agents were made by the treating rheumatologist as part of routine rheumatoid arthritis management, and detailed information regarding the rationale for each therapeutic modification was not always available. Furthermore, there is currently no established evidence regarding the optimal duration of upadacitinib therapy for scleritis, and it remains unclear whether long-term treatment over multiple years is necessary.

## Conclusion

This case suggests that upadacitinib, a selective JAK1 inhibitor, may be associated with clinical improvement in necrotizing scleritis refractory to multiple biologic therapies. By targeting intracellular cytokine signaling through the JAK-STAT pathway, upadacitinib may provide broader immunomodulation than biologics directed against single cytokines. However, given the single-case nature of this report, the findings should be interpreted with caution. Further prospective studies and larger case series are required to better define the efficacy, safety, and optimal treatment duration of JAK inhibitors in refractory scleritis and other autoimmune ocular inflammatory diseases.

## Data Availability

Data sharing is not applicable to this article as no datasets were generated or analyzed. All relevant clinical information supporting the findings of this report is included in the article.
